# Editorial: New drugs for treating COVID-19 cancer patients

**DOI:** 10.3389/fendo.2023.1151942

**Published:** 2023-03-09

**Authors:** Keng Po Lai, Rong Li, Ka Wu, William Ka Fai Tse

**Affiliations:** ^1^ Key Laboratory of Environmental Pollution and Integrative Omics, Education Department of Guangxi Zhuang Autonomous Region, Guilin Medical University, Guilin, China; ^2^ Department of Pharmacy, The Second People’s Hospital of Nanning City, The Third Affiliated Hospital of Guangxi Medical University, Nanning, China; ^3^ Laboratory of Developmental Disorders and Toxicology, Center for Promotion of International Education and Research, Faculty of Agriculture, Kyushu University, Fukuoka, Japan

**Keywords:** traditional Chinese medicine, SARS-CoV-2, vaccination, drug development, cancer patient

Since 2019, the world population has been affected by the coronavirus pandemic (COVID-19). Although many COVID-19 vaccines are available, variants of SARS-CoV-2 have reduced their effectiveness. Prospective cohort studies have demonstrated that COVID-19 patients with underlying malignancies such as cancer have a higher mortality rate than those without them, resulting in an urgent need to identify drugs for cancer patients infected with COVID-19. Current research findings have shown that integrated traditional Chinese and Western medicine can be effective in preventing COVID-19 and relieving its clinical symptoms. Some traditional Chinese medicines (TCMs) were officially recommended by the National Medical Products Administration to treat COVID-19. They are commonly used for chronic clinical disorders, including malignant cancers, and can potentially be used for the treatment of cancers associated with COVID-19. Pharmacological studies have suggested that TCM is effective for the treatment of COVID-19 *via* its host-directed regulation and certain antiviral effects. The identification of new effective treatments and a better understanding of the molecular mechanisms of new drugs for treating COVID-19 and different cancer types could provide a new insight into this field. Moreover, immunologic deficiency in cancer patients could increase the severities of COVID-19 infections. Drugs that are used to improve the immune system could be another direction for developing the target therapies.

The current Research Topic is to be found in the Cancer Endocrinology section of the Frontiers in Endocrinology journal; a sum of eight contributing articles (five research articles and three review articles) have been included, and we would like to summarize the issue into the three following major directions.

## Vaccination and safety

Joudi et al. performed a cohort study to address the immunogenicity and safety issue of using the SARS-CoV-2 vaccine (BBIBP-CorV) on breast cancer patients. The study included 160 breast cancer patients, who were followed up for three months after the vaccination. They reported that 85.7% and 87.4% of the patients were positive for SARS-CoV-2 Anti-Spike IgG and SARS-CoV2 Anti-RBD IgG, respectively, with a reduced rate of COVID-19 infection in the study period.

Javadinia et al. performed a meta-analysis on publications regarding the safety and efficacy of COVID-19 vaccines in cancer patients from 1/1/2019 to 30/11/2021. A total of 28 articles passed all the selection criteria and could be used. They concluded that COVID-19 vaccination in patients with malignancies is safe and tolerable regardless of the type, and the reported systemic and locoregional side effects were given a maximum rank of grade II.

## Drug delivery system and therapeutic strategy

Prostate cancer has features with high immunogenicity that make it difficult to be treated by immune checkpoint blocking therapy. A survey reported that prostate cancer is responsible for one-third of COVID-19-infected male cancer patients. Li et al. developed a delivery platform by combining the use of Zn^2+^ with a doxorubicin (DOX)-loaded black phosphate nanometer with photothermal therapy to target prostate cancer cells. Such a system could induce immunogenic cell death and dendritic cell maturation in the cancer cells, which may be further developed into a form of prostate cancer vaccine to activate the immune response.

Breast cancer patients possess the estrogen receptor positive (ER^+^) feature, and thus antiestrogen therapies are used to treat them. Currently, selective estrogen receptor modulators (SERMs) are suggested to be an effective treatment for breast cancer patients with COVID-19 due to their anti-ER and antiviral effects. Hu et al. reviewed the topic by summarizing the background of estrogen receptors and endocrine therapy. They ended the article by reviewing both the beneficial effects and side effects of the two common SERMs (raloxifene and tamoxifen) on breast cancer patients.

Qu et al. presented another review on the therapeutic approaches to thyroid cancer and COVID-19. SARS-CoV-2 infection has been reported to induce thyroid diseases, and numerous inhibitors have been suggested to treat thyroid cancer. The authors summarized the related works and suggested that target therapies such as multikinase inhibitors and immunotherapy may be used to improve the therapy and management of thyroid cancer patients with COVID-19.

## Network pharmacology

Ferulic acid has been shown to perform various biological actions, such as those of antibacterial, antiviral, and antineoplastic natures. A network pharmacology study with a bioinformatics analysis was conducted by Pang et al., who identified the signal transducer and activator of transcription 3 (STAT3), mitogen-activated protein kinase 1 (MAPK1), and phosphoinositide-3-kinase regulatory subunit 1 (PIK3R1) as the pharmacological targets of ferulic acid in treating the osteosarcoma by regulating various anticancer and antiviral signaling pathways.

Mogroside V (a bioactive ingredient from *Siraitia grosvenorii*) is used to prevent asthma and has shown inhibitory effects on certain cancers. Another report by Li et al., using a similar approach, has suggested that mogroside V shares common targets with COVID-19 and ovarian cancer. Vascular endothelial growth factor A (VEGFA) was found to be a core protein in the bioinformatics analysis. The study identified ten core targets of mogroside V against COVID-19 and ovarian cancer. Those targets were found to play roles in cytokine activities, the HIF-1 signaling pathway, and Th17 cell differentiation that are enhanced in cancer patients. They demonstrated that the mogroside V could form favorable hydrogen bonding with the VEGFA in the study and suggested its potential use in ovarian cancer patients.

The third example given in this special issue is the use of luteolin on prostate cancer. Luteolin is an antioxidant flavonoid that is widely found in plants, which has been shown to have anticancer activity. Ye et al. first searched for the differentiated expressed genes in prostate cancer patients and further applied them to identify the common targets of luteolin. Eighteen intersection targets that are related to various signaling pathways such as HIF-1, TNF, and AMPK were found. The authors further reported that the two core targets, myeloperoxidase and FOS, could bind with luteolin and thus act as a potential drug for prostate cancer and COVID-19.

In conclusion, this issue has provided a platform for both clinical and basic science researchers to discuss the challenges of treating cancer patients infected with COVID-19. Various approaches have been applied to overcome such challenges. Network pharmacology is one of the most powerful methods to predict the potential binding site and relative functions of the target. However, it should be noted that there are limitations to such *in silico* studies. Biological experiments must be performed to understand the downstream actions and molecular mechanisms. Nevertheless, such a method provides supportive data to reduce the time for reaching targets in these contemporary matters.

## Author contributions

All authors listed have made a substantial, direct, and intellectual contribution to the work and approved it for publication.

